# A Possible Role for End-Stopped V1 Neurons in the Perception of Motion: A Computational Model

**DOI:** 10.1371/journal.pone.0164813

**Published:** 2016-10-14

**Authors:** Parvin Zarei Eskikand, Tatiana Kameneva, Michael R. Ibbotson, Anthony N. Burkitt, David B. Grayden

**Affiliations:** 1 NeuroEngineering Laboratory, Dept Electrical & Electronic Engineering, The University of Melbourne, Parkville, VIC 3010, Australia; 2 NICTA Victorian Research Laboratory, Parkville, VIC 3010, Australia; 3 National Vision Research Institute, Australian College of Optometry, Carlton, VIC 3053, Australia; 4 Centre for Neural Engineering, The University of Melbourne, Parkville, VIC 3030, Australia; McGill University Department of Physiology, CANADA

## Abstract

We present a model of the early stages of processing in the visual cortex, in particular V1 and MT, to investigate the potential role of end-stopped V1 neurons in solving the aperture problem. A hierarchical network is used in which the incoming motion signals provided by complex V1 neurons and end-stopped V1 neurons proceed to MT neurons at the next stage. MT neurons are categorized into two types based on their function: integration and segmentation. The role of integration neurons is to propagate unambiguous motion signals arriving from those V1 neurons that emphasize object terminators (e.g. corners). Segmentation neurons detect the discontinuities in the input stimulus to control the activity of integration neurons. Although the activity of the complex V1 neurons at the terminators of the object accurately represents the direction of the motion, their level of activity is less than the activity of the neurons along the edges. Therefore, a model incorporating end-stopped neurons is essential to suppress ambiguous motion signals along the edges of the stimulus. It is shown that the unambiguous motion signals at terminators propagate over the rest of the object to achieve an accurate representation of motion.

## Introduction

**Fig 1:**
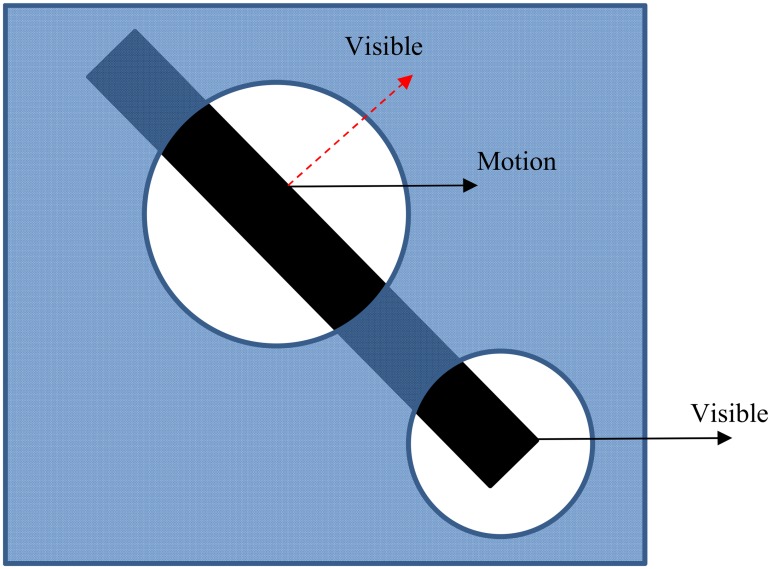
A schematic explanation of the aperture problem. The bar is moving to the right. The component of motion that is parallel to the edge of the bar is not visible in the upper aperture because there is no change in the contrast in this direction. Therefore, it seems that the bar is moving in a direction that is perpendicular to the edge of the bar (arrow labeled “visible”). The correct direction of motion can be estimated when the end-points of the bar are seen through an aperture as shown in the lower aperture.

Visual information processing in the cortex begins in the primary visual cortex (V1) of the occipital lobe, which receives its input from the retina via the dorsal lateral geniculate nucleus (LGN) [1]. Information from V1 is sent to higher brain regions through two cortical pathways: the dorsal and ventral pathways [1]. The main role of the dorsal pathway is to determine the spatial location and motion of stimuli, while the ventral pathway is specialized for processing form and color information. Processing of motion information starts in V1 but the receptive fields of the neurons in this area are very small, with diameters in the central visual field of less than one degree. Due to these small receptive fields, the neurons can only measure local motion signals; i.e., the component of the motion that moves orthogonal to the orientation of an edge. Fig 1 illustrates this effect: any component of the motion parallel to the edge is not visible because of the invariance of the contrast in this direction. Measuring only one component of the motion results in an ambiguity called the “aperture problem” [2,3]. To overcome this problem, these initial motion signals are fed to neurons with larger receptive fields in an early part of the dorsal pathway, known as area MT (also V5).

There is experimental evidence that MT neurons play a critical role in suppressing ambiguous motion information [4–6]. Various computational models of MT neurons have been proposed to show how these neurons deal with the aperture problem. A well-known model, referred to as the intersection of constraints, uses the local motion of two edges to compute the global motion by finding the intersection of all of the detected local motions [7]. Simoncelli and Heeger (1998) showed that MT neurons might use this method to extract two-dimensional motion signals [8]. Other models strengthen unambiguous motion signals relative to the ambiguous feature signals [9–11]. In these models, there is no way to propagate the feature tracking signals to estimate the accurate motion direction in a single stage. Therefore, another stage is necessary, which is known as a motion-grouping network [10]. In this model, there is competition between motion signals received from V1. Neurons with a similar direction preference, selected as winning cells, send feedback activity to suppress other directions. Therefore, a model of the medial superior temporal (MST) area, which is the next anatomical stage after MT, is essential in these models to solve the aperture problem by propagating motion information from the terminators. In addition, there are no inhibitory connections to limit the propagation of the activities of MT neurons beyond the border of the stimulus, where there is no motion [10–13].

Liden and Pack (1999) proposed a model of MT neurons that propagates motion information along the stimulus. However, in this model, there is no strategy to discriminate the unambiguous motion information of the terminators from the ambiguous signals resulting from the aperture problem [14]. Therefore, the neighboring neurons along the edges that are active simultaneously over a larger area dominate the other neurons. This results in suppression of the unambiguous activity of the neurons responding to the terminators, which only cover a small area of the stimulus, before the propagation of their activity to other regions. Moreover, the activity of V1 neurons in this model is computed from the spatial correlation between subsequent frames. Therefore, the neurons have their highest level of activity where there is no motion (i.e., in the stimulus background). In their proposed model, there is also no mechanism to detect the border of the stimulus. Therefore, the activities of the V1 neurons that respond to the background of the stimulus dominate over other neurons that represent motion information before the suppression of their activity by inter-directional inhibition between neurons.

In another approach proposed by Bayerl and Neumann (2004), combinations of feedback connections from area MT and lateral inhibition are used to suppress the ambiguous responses of neurons [15]. The result of this model suggests that V1 neurons have ambiguous perception of motion at the early stages of their response and this ambiguity is resolved through feedback connections from MT neurons, which have less ambiguous activity because of their extended receptive fields. Although this is an interesting approach, there is no biological evidence to represent the propagation of unambiguous motion information to other V1 neurons over time. The temporal evolution of the response of MT neurons has been observed experimentally by Pack and Born (2001), whose results show that MT neurons overcome their ambiguous representation of motion with a delay of 200ms after the initial onset of the stimulus [5]. However, there is no biological evidence to show that standard V1 neurons follow the same temporal pattern to deal with the aperture problem.

Neuro-physiological experiments have revealed a subset of orientation-selective neurons in V1 called “end-stopped cells” that have inhibitory regions outside the excitatory receptive field area. As an optimally oriented bar increases in length between the inhibitory zones, the response of an end-stopped cell increases, but when the bar extends into the inhibitory zones, its response is suppressed, such that the cell is strictly length selective [16]. In cases where cells are both direction selective and end-stopped, the cells respond maximally to the end-points of bars, i.e. “terminators” [17] (Fig 1). As the terminators are two-dimensional, the estimated direction of motion is accurate compared to the one-dimensional regions in which the measured direction of motion is perpendicular to the edge of the object. End-stopped neurons respond only to the motion information of terminators that cover a small area of the stimulus. Therefore, a large amount of the motion information signals transmitted to the MT neurons are ambiguous motion signals from the standard complex V1 neurons. It is the role of MT neurons to propagate unambiguous motion information from the terminators to the other areas of the input stimulus. Therefore, solving the problem is a two-stage process that is initiated in the primary area of V1 with the activity of end-stopped neurons and completes with the neurons in MT by integrating the received local motion signals from V1.

Experiments performed to explore the temporal dynamics of end-stopped cells revealed that the end-stopping takes some time to develop because it involves an interaction between the center and surround of the neuron’s receptive field [17]. Therefore, end-stopping is a dynamic behavior. For example, at an early stage of stimulus presentation, neurons respond well to a long bar but, after about 20–30 ms terminator inhibition suppresses the response and the neurons begin to respond preferentially to motion at the end-points of the bar [17]. Based on neuro-physiological experiments, many neurons in V1 have some end-stopping (suppressive surround) characteristics, but it is not clear how this feature might be involved in overcoming the aperture problem [18,19].

The direction-selective end-stopped neurons in V1 provide initial motion signals to MT neurons [20]. Studies have shown that the receptive fields of MT neurons have a very sophisticated structure. The neurons do not respond directly to stimulation in the area outside their excitatory receptive fields. However, their responses are strongly influenced by the differences between the stimulus inside and immediately outside their excitatory receptive field, the latter known as a “silent surround” [3,21–23]. This center-surround interaction is mostly antagonistic, which helps to detect image segmentation [22,24]; i.e., when the stimuli in the receptive field and surround move in different directions, the surround has an additive excitatory effect on the response of the neuron. Conversely, when the stimuli move in the same direction, the activity in the surround suppresses the activity generated by motion in the neuron’s excitatory receptive field. While the antagonistic influence of the surround has been thoroughly investigated, integrative influences of the surround have only been found for selected stimuli [25]. These integrative effects, where present, may help to overcome the aperture problem.

Here, we investigate the potential role of V1 end-stopped cells for accurate estimations of motion direction. Based on the observations made by Huang and Albright (2007), and inspired by a neural network proposed by Liden and Pack (1999), we developed a model for the perception of motion that is performed in two stages [14,25]. In the first stage, there are two sets of V1 neurons: end-stopped neurons and regular complex neurons with no end-stopping properties. These V1 neurons provide input to the second stage of the network, which we model as being representative of processing by MT neurons. The MT neurons are divided into two different sets based on their function: integration and segmentation. The role of segmentation neurons is to detect discontinuities in the input image, while integration neurons gather all of the local motion signals in V1 to estimate the correct direction of motion, thus overcoming the aperture problem. In our model, integration and segmentation neurons interact with each other through different types of connections to estimate the direction of the moving object.

Our model proposes some advantages over other similar computational methods. The inclusion of end-stopped neurons in our model assists MT neurons to weight more strongly the received unambiguous motion signals from the terminators over the other areas of the input stimulus to overcome the aperture problem, which is in contrast to the model proposed by Liden and Pack (1999) [14]. Furthermore, the aperture problem in our model is thoroughly solved at the intermediate stage (MT) of the visual cortex and the participation of the neurons in higher levels, such as MST, is not essential, unlike other approaches [10–13] where a model of MST neurons is essential for the propagation of the motion signals from the terminators along the whole of the stimulus. The characteristics of our proposed model in both areas V1 and MT are biologically realistic. The common model of motion energy filters used in V1 matches available neurophysiological data, but the modeled V1 neurons by Liden and Pack (1999) have the highest level of activity where there is no motion, as they are a correlation-based model with higher output values where there is no variation in the stimulus [14]. The mechanism used to propagate the motion information by Bayerl and Neumann (2004) also does not accord with the current neurophysiological data that shows the activity of V1 neurons do not evolve over time to disambiguate motion information [15]. In contrast, our model uses the interconnection between MT neurons to propagate motion information from the terminators and this propagation does not have any effect on the activity of V1 neurons. The experiments by Pack and Born (2001) provide evidence for this delayed disambiguation of the activity of MT neurons [5].

## Methods

We propose a neural model that can determine the true direction of motion by replicating the response properties of several known neuronal types in brain areas V1 and MT and the interconnections between them. The initial motion signals are computed through spatiotemporal filters with different orientation tuning. The filters aim to replicate the response properties of neurons in V1. There are neurons in V1 that have end-stopping features that provide unambiguous motion signals to MT neurons. The resulting activity of the end-stopped neurons is passed through excitatory connections to MT neurons for further processing. Based on the characteristics of the receptive field surround of the MT neurons, they integrate or segregate the local motion signals received from V1 neurons to detect the borders of the input image and overcome the aperture problem.

**Fig 2:**
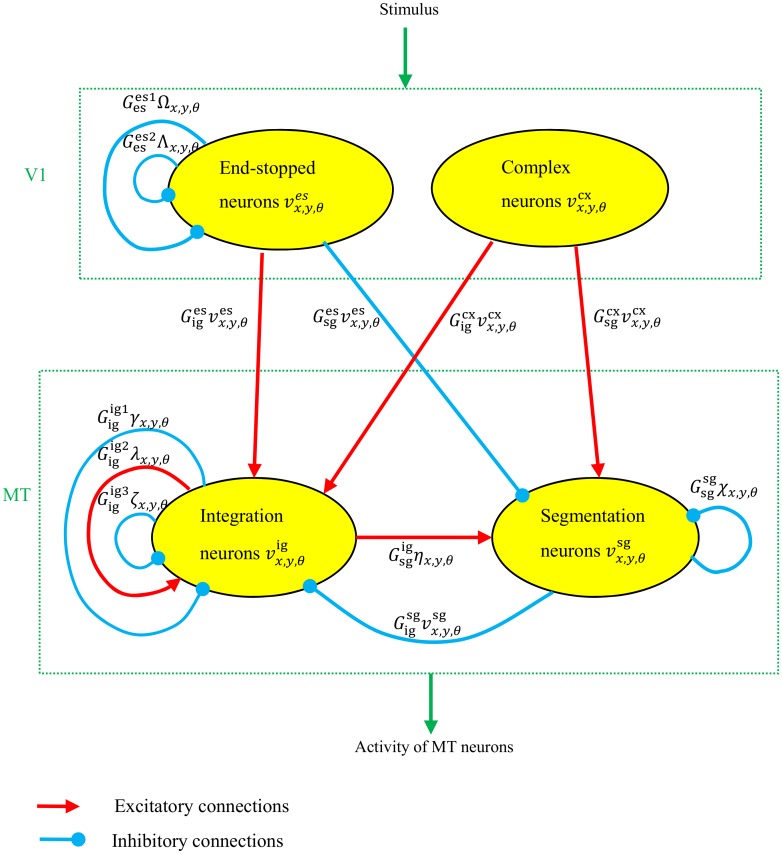
The interconnections between neurons in V1 and MT. The interconnections represented by red (excitatory) and blue (inhibitory) arrows, respectively. Integration neurons receive inputs from both sets of complex and end-stopped cells in V1. They also receive inhibitory connections from segmentation cells. Segmentation cells receive excitatory input from complex cells and are inhibited by end-stopped cells. They also receive a conditional inhibitory connection from integration cells when the neurons in the receptive field center and surround are active. The connection parameters and variables are explained in the text and in Tables 1 and 2.

Fig 2 is a schematic diagram of the excitatory and inhibitory connections between neurons in V1 and MT employed in our model. Based on surround suppression, MT neurons are divided into two categories: integration and segmentation neurons. These neurons interact with each other to propagate unambiguous motion signals from the terminators along the whole of the object. Table 1 is a summary of the model parameters. In these parameters, ‘ig’ and ‘sg’ are short forms used to describe integration and segmentation cells, respectively, and ‘cx’ and ‘es’ are short forms for complex and end-stopped neurons, respectively.

**Table 1:** Variables used in the model.

Description	Parameter
Activity of complex neuron	*v*^cx^
Activity of end-stopped neuron	*v*^es^
Activity of integration neurons in MT	*v*^*ig*^
Activity of segmentation neurons in MT	*v*^sg^
Inter-directional inhibitory connections between end-stopped neurons	Ω
Long-range inhibitory connections between end-stopped neurons	Λ
Excitatory input of end-stopped neurons	Γ
Inter-directional inhibitory connection between integration neurons	*γ*
Long-range inhibitory connection between integration neurons	ζ
Excitatory connection from integration to segmentation neurons	*η*
Inhibitory connection from integration neurons as the result of center-surround antagonism	*χ*
Preferred direction of neurons	*θ*
Horizontal location index	*x*
Vertical location index	*y*
Location index of the area that a neuron receives surround suppression	Π
Location index of the area that a neuron receives long-range inhibition	Φ
Motion energy in the specific direction	*r*
Motion energy in the opposite direction	*l*
Input stimulus	*I*

### Complex V1 neurons

The activities of complex neurons in V1 are computed using motion energy filters [26]. This model is composed of oriented spatiotemporal filters that generate initial motion energy signals. The spatial filters are Gabor functions with sine and cosine phase. The width of the filter covers four pixels of the stimulus. The temporal filter is a multi-stage low-pass filter expressed as

$$g_{n}(t)={\left(t/\tau_{g}\right)}^{n}\text{exp}(-t/\tau_{g})\;\left[\frac{1}{n!}-\frac{{\left({t/\tau }_{\text{g}}\right)}^{2}}{(n+2)!}\right].$$ (1)

To simulate two temporal filters with different delays, *n* takes two values, *n* = 6 and *n* = 9, and *τ*_*g*_ is the time constant of the filter. The output from these spatial and temporal filters are combined and squared to obtain separable spatiotemporal filters. The result is a good approximation of the motion energy in a direction, *r*_*x*, *y*, *θ*_ (*t*), and the opposite direction, *l*_*x*, *y*, *θ*_ (*t*),

$$\begin{array}{ll} r_{x,y,\theta }^{}(t)= & \left(-I_{x,y}(t)*\text{sin}(2\pi fx_{\theta }^{})\text{ exp}(\frac{-x_{\theta }^{2}}{\sigma_{x}^{2}}+\frac{y_{\theta }^{2}}{\sigma_{y}^{2}})*g_{6}(t\right)\\ & +(I_{x,y}(t)*\text{cos}(2\pi fx_{\theta })\text{ exp}(\frac{-x_{\theta }^{2}}{\sigma_{x}^{2}}+\frac{y_{\theta }^{2}}{\sigma_{y}^{2}}))*g_{9}(t){)}^{2}\\ & +(I_{x,y}(t)*\text{sin}(2\pi fx_{\theta })\text{ exp}(\frac{-x_{\theta }^{2}}{\sigma_{x}^{2}}+\frac{y_{\theta }^{2}}{\sigma_{y}^{2}})*g_{9}(t)\\ & +(I_{x,y}(t)*\text{cos}(2\pi fx_{\theta })\text{ exp}(\frac{-x_{\theta }^{2}}{\sigma_{x}^{2}}+\frac{y_{\theta }^{2}}{\sigma_{y}^{2}}))*g_{6}(t){)}^{2}, \end{array}$$ (2)

$$\begin{array}{ll} l_{x,y,\theta }^{}(t)= & \left(I_{x,y}(t)*\text{sin}(2\pi fx_{\theta })\text{ exp}(\frac{-x_{\theta }^{2}}{\sigma_{x}^{2}}+\frac{y_{\theta }^{2}}{\sigma_{y}^{2}})*g_{6}(t\right)\\ & +(I_{x,y}(t)*\text{cos}(2\pi fx_{\theta })\text{ exp}(\frac{-x_{\theta }^{2}}{\sigma_{x}^{2}}+\frac{y_{\theta }^{2}}{\sigma_{y}^{2}}))*g_{9}(t){)}^{2}\\ & +(-I_{x,y}(t)*\text{sin}(2\pi fx_{\theta })\text{ exp}(\frac{-x_{\theta }^{2}}{\sigma_{x}^{2}}+\frac{y_{\theta }^{2}}{\sigma_{y}^{2}})*g_{9}(t)\\ & +(I_{x,y}(t)*\text{cos}(2\pi fx_{\theta })\text{ exp}(\frac{-x_{\theta }^{2}}{\sigma_{x}^{2}}+\frac{y_{\theta }^{2}}{\sigma_{y}^{2}}))*g_{6}(t){)}^{2}, \end{array}$$ (3)

where *I*_*x*, *y*_ (*t*) is the input stimulus at the spatial location (*x*, *y*), *f* is the spatial frequency of the sinusoidal carrier, (*x*_*θ*_, *y*_*θ*_) represents the oriented coordinate in direction *θ*, and *σ*_*x*_ and *σ*_*y*_ are standard deviations. The symbol * represents convolution. To extract movement in eight directions, four motion energy filters with orientations 0, *π*/4, *π*/2, and 3*π*/2 are used. For example, for a filter oriented at 0°, a positive value of its output represents a rightward motion and a negative value indicates leftward motion. The outputs of model complex neurons in V1 are the normalized values of the output of motion energy filters according to

$$v_{x,y,\theta }^{\text{cx}}(t)=\frac{r_{x,y,\theta }^{}}{\sqrt{r_{x,y,\theta }^{2}+l_{x,y,\theta }^{2}}},$$ (4)

$$v_{x,y,\theta -\pi }^{\text{cx}}(t)=\frac{l_{x,y,\theta }^{}}{\sqrt{r_{x,y,\theta }^{2}+l_{x,y,\theta }^{2}}},$$ (5)

where $v_{x,y,\theta }^{\text{cx}}$ is the activity of the complex V1 neuron at location (*x*, *y*) that is selective to direction *θ*. The resulting neurons respond strongly to motion in their preferred direction and have no activity when there is no movement in their preferred direction.

### End-stopped neurons

End-stopped neurons are modelled using lateral inhibition between neighboring neurons (in the receptive field surround) that are selective to the same direction as the central end-stopped cell. This inhibition is active only if the neighboring neurons of a cell have a level of activity above a set threshold. The value of the inhibition decreases in a Gaussian form with increasing distance from the central neuron. The activities of the end-stopped cells are computed according to

ddtvx,y,θes(t)=(1−vx,y,θes(t))(Gescx1vx,y,θcx(t))   −vx,y,θes(τes+Gescx2 Γ(t)x,y,θ+Geses1Ωx,y,θ(t−Tes)+Geses2Λx,y,θ(t−Tes)), (6)

where $v_{x,y,\theta }^{\text{es}}$ is the activity of an end-stopped cell selective to direction *θ* located at the coordinate (*x*, *y*), $v_{x,y,\theta }^{\text{cx}}$ is the activity of the complex neuron in the same location and direction, *τ*_*es*_ is a decay rate, and $G_{\text{es}}^{\text{cx1}}$, $G_{\text{es}}^{\text{cx2}}$, $G_{\text{es}}^{\text{es1}}$, and $G_{\text{es}}^{\text{es2}}$ are gains. *Γ*_*x*, *y*, *θ*_ is the inhibition that the neuron receives from complex neurons when the activity of the neighboring complex neurons are above a certain threshold, *ρ*_cx_,

$$\Gamma_{x,y,\theta }^{}=\left\{ \begin{array}{l} \sum_{\begin{array}{l} i=-8 \end{array}}^{8}\sum_{\begin{array}{l} j=-8 \end{array}}^{8}\mu_{i,j}\;v_{x+i,y+j,\theta }^{\text{cx}}\;{v_{\text{x}+\text{i},\text{y}+\text{j,}\theta }^{\text{cx}}|}_{i=-3,j=-3}^{i=3,j=3}>\rho_{\text{cx}}\\ 0\text{ otherwise,} \end{array}\right.$$ (7)

where *μ*_*i*, *j*_ is the inhibitory connectivity matrix, which has a discretized Gaussian shape.

There is also an inter-directional inhibition between end-stopped neurons selective to different directions. The value of this inhibition depends on the level of the activity of the V1 complex neurons selective to other directions,

$$\Omega_{x,y,\theta }=\sum_{\phi =\theta }v_{x,y,\phi }^{\text{cx}}.$$ (8)

The role of these inhibitory connections is more prominent when there is no aperture problem. This happens when the difference in the orientation of the stimulating bar and the direction of the movement is 0° or 90°. End-stopped neurons also receive long-range inhibitory connections from other neighboring end-stopped neurons that are selective to other directions,

$$\Lambda_{x,y,\theta }=\sum_{\phi \neq \theta }\sum_{i=-3}^{3}\sum_{j=-3}^{3}v_{x+i,y+j,\theta }^{\text{es}}.$$ (9)

These inhibitory inter-directional connections between end-stopped neurons are applied with a delay of 60ms to permit the development of activity in these neurons from complex V1 neurons before enforcing inhibitory connections between them.

### MT integration neurons

**Fig 3:**
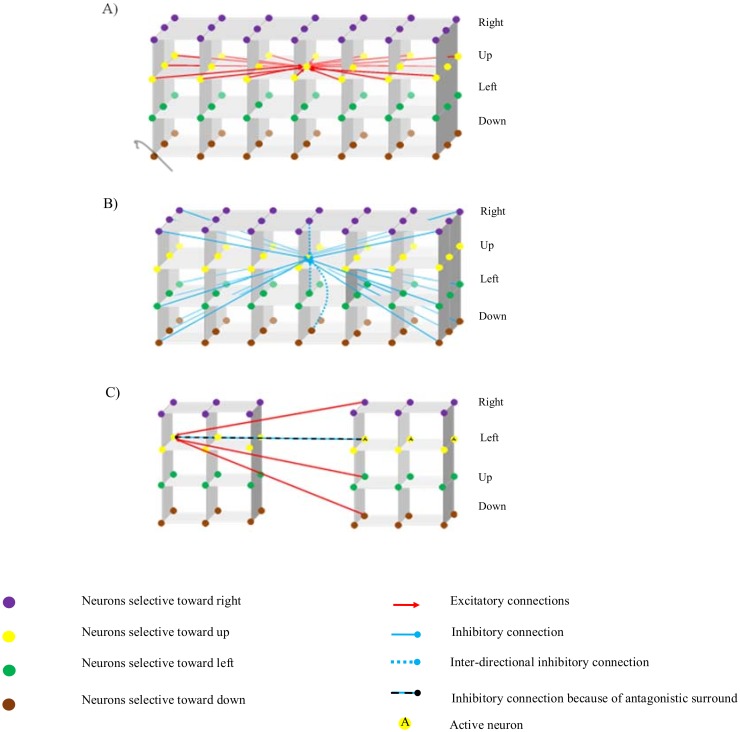
Schematic diagram of interconnections between neurons in MT. Nodes with the same color are neurons selective to the same direction. Neurons in columns have the same spatial location but different direction selectivity. A) Red arrows indicate the excitatory connections from neighboring integration neurons with the same directional preference. Neurons receive excitatory inputs from nearby integration neurons and neurons in the surround. B) A schematic diagram of inhibition from distant neighboring neurons selective to different directions. Solid blue arrows represent these long-range inhibitory connections. In addition, integration neurons receive inter-directional inhibition from neurons selective to different directions at the same spatial location; the dotted blue arrows represent these interconnections. C) The inter-connection between integration and segmentation neurons in MT. Segmentation neurons receive excitatory connections from integration neurons with different directional preferences at the same spatial location. They also receive inhibition from segmentation cells when motion in the center and surround of the receptive field of a cell is in the same direction.

The interconnections between neurons in MT are shown in Fig 3. Interactions between integration neurons have three components: (1) excitation from neighboring neurons selective to the same direction, (2) inter-directional inhibition between neurons at the same spatial location, and (3) long-range inhibition from distant neighboring neurons. The behavior of the integration neurons, $v_{x,y,\theta }^{\text{ig}}$, is determined by

$$\frac{dv_{x,y,\theta }^{\text{ig}}(t)}{dt}=h\left(\begin{array}{l} G_{\text{ig}}^{\text{cx}}v_{x,y,\theta }^{\text{cx}}(t)+G_{\text{ig}}^{\text{es}}v_{x,y,\theta }^{\text{es}}(t)+G_{\text{ig}}^{\text{ig2}}\lambda_{x,y,\theta }^{}(t)-G_{\text{ig}}^{\text{ig1}}\gamma_{x,y,\theta }^{}(t-T^{\text{ig}})\\ -G_{\text{ig}}^{\text{ig3}}\zeta_{x,y,\theta }^{}(t-T^{\text{ig}})-G_{\text{ig}}^{\text{sg}}v_{x,y,\theta }^{\text{sg}}(t)-\tau_{\text{ig}}v_{x,y,\theta }^{\text{ig}}(t) \end{array}\right),$$ (10)

where *λ*_*x*, *y*, *θ*_ is excitation from neighboring neurons, *γ*_*x*, *y*, *θ*_ is inter-directional inhibition, and *ζ*_*x*, *y*, *θ*_ is long-range inhibition. These neurons receive excitatory input from V1 complex and end-stopped neurons, $v_{x,y,\theta }^{\text{cx}}$ and $v_{x,y,\theta }^{\text{es}}$, respectively. They also receive inhibitory input from segmentation neurons, $v_{x,y,\theta }^{\text{sg}}$. Finally, *h*() is a piece-wise linear saturation function that keeps the level of activity within a specified range (between 0 and 1):

$$h(x)=\{\begin{array}{ll} 1 & \text{if }x\geq 1\\ x & \text{if 0}\leq \text{x}<\text{1}\\ 0 & \text{if x}<\text{0} \end{array}$$ (11)

The remaining parameters have constant values (Table 2) that were determined using the genetic algorithm described below.

**Table 2:** The constant parameters used in the model, their values, and their units.

Description	Parameter	Value	Unit
Connection strength of input to the end-stopped neurons	$G_{\text{es}}^{\text{cx}1}$	2	—
Connection strength of inhibitory connections on end-stopped neurons	$G_{\text{es}}^{\text{cx}2}$	3	—
Connection strength of inter-directional inhibitory connections between end-stopped neurons	$G_{\text{es}}^{\text{es}1}$	1	—
Connection strength of long-range inhibitory connections between neighboring end-stopped neurons	$G_{\text{es}}^{\text{es}2}$	0.5	—
Connection strength of complex V1 neurons to integration neurons	$G_{\text{ig}}^{\text{cx}}$	0.3	—
Connection strength of end-stopped V1 neurons to integration neurons	$G_{\text{ig}}^{\text{es}}$	1	—
Connection strength of complex V1 neurons to segmentation neurons	$G_{\text{sg}}^{\text{cx}}$	1	—
Connection strength of end-stopped V1 neurons to segmentation neurons	$G_{\text{sg}}^{\text{es}}$	1	—
Connection strength of excitatory connections to integration neurons	$G_{\text{ig}}^{\text{ig}2}$	0.1	—
Connection strength of inter-directional inhibitory connections	$G_{\text{ig}}^{\text{ig}1}$	0.741	—
Connection strength of long range inhibitory connections	$G_{\text{ig}}^{\text{ig}3}$	0.1	—
Connection strength of inhibition from segmentation neurons	$G_{\text{ig}}^{\text{sg}}$	1	—
Connection strength of excitation from integration on segmentation neurons	*$G_{\text{sg}}^{\text{ig}1}$*	0.7	—
Connection strength of surround suppression on segmentation neurons	$G_{\text{sg}}^{\text{sg}}$	1	—
Number of neurons at each location selective to different directions	*N*	8	—
Threshold on the activity of segmentation neurons	*ρ*_sg_	0.01	—
Threshold on the activity of integration neurons	*ρ*_ig_	0.1	—
Threshold on the difference between the activity of the integration neurons	$\rho_{\text{ig}}^{\text{ig}}$	0.01	—
Threshold on the activity of end-stopped neurons	*ρ*_es_	0.008	—
Decay rate of the activity of integration neurons	*τ*_ig_	0.101	—
Decay rate of the activity of segmentation neurons	*τ*_sg_	0.101	—
Decay rate of the activity end-stopped neurons	*τ*_es_	0.01	—
Simulation time step	Δ*t*	0.1	ms
Time constant of the temporal filter	*τ*_g_	0.01	ms
Time delay of inhibition between end-stopped neurons	*T*^es^	6	ms
Time delay of inhibition between MT integration neurons	*T*^*ig*^	6	ms
Spatial frequency	*f*	1.1	cyc/deg
Standard deviation of horizontal spatial Gaussian filter	*σ*_*x*_	0.5	—
Standard deviation of vertical spatial Gaussian filter	*σ*_*y*_	0.5	—

The functional role of the excitatory connections is to propagate unambiguous signals from the terminators along the object. To overcome the aperture problem, neurons receive excitation only from neighboring neurons with higher levels of activity; i.e.,$\;v_{x+i,y+j,\theta }^{\text{ig}}-v_{x,y,\theta }^{\text{ig}}>\rho_{\text{ig}}^{\text{ig}}$. This interaction between MT neurons is modelled as

$$\lambda_{x,y,\theta }^{}=\left\{ \begin{array}{l} \sum_{i=-6}^{6}\sum_{j=-6}^{6}v_{x+i,y+j,\theta }^{\text{ig}}\;H(\rho_{\text{sg}}-v_{x,y,\theta }^{\text{sg}}),\text{ if }v_{x+i,y+j,\theta }^{\text{ig}}-v_{x,y,\theta }^{\text{ig}}>\rho_{\text{ig}}^{\text{ig}}\\ 0,\text{ otherwise, } \end{array}\right.$$ (12)

where (*x+i*, *y+j*) is a location index and *H*() is the Heaviside step function. When segmentation neurons have a value above the threshold, *ρ*_*sg*_, they prevent integration neurons from receiving this excitatory input. This means that segmentation neurons limit the activity of integration neurons at the discontinuities of the input stimulus, where they are activated by effectively shunting excitation from neighboring integration neurons.

**Fig 4:**
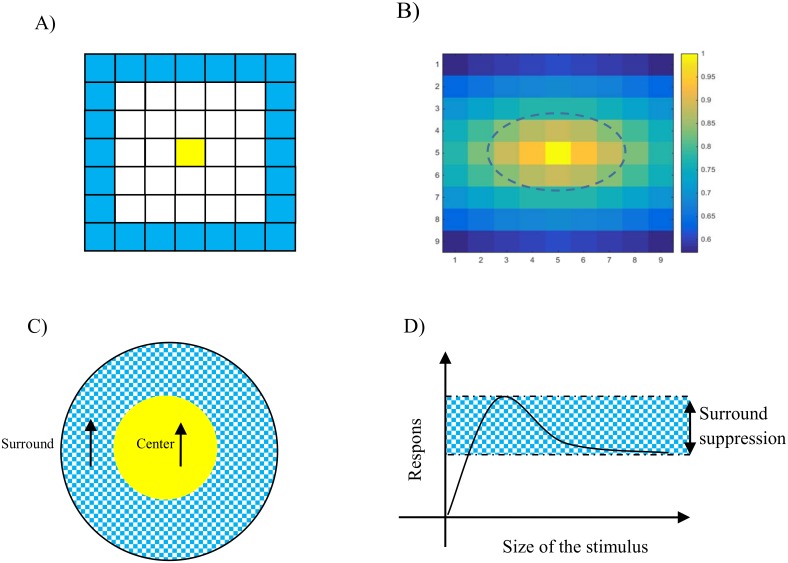
Illustration of lateral inhibition in integration and segmentation neurons. A) The area from which an integration neuron, denoted in yellow, receives long range inhibition is represented by blue squares. B) The spatial extent of the surround of the receptive fields of MT neurons covers around nine pixels of the input stimulus. The area from which the segmentation neuron receives surround suppression is the outside of the border represented by the dashed line. The receptive field of the neuron is modelled as a Gaussian filter in which the amplitude decreases gradually from the center of the receptive field, with the yellow color representing the highest value. C) The circle shows the excitatory receptive field of a segmentation MT neuron and its surround area. According to experimental data, when motion in the center and surround of the neuron are in the same direction, the response of the segmentation cell is suppressed. D) Illustration of the effect of surround suppression, where increasing the size of the input stimulus, such that the center and surround are stimulated simultaneously, the activity of a segmentation MT neuron experiences a suppression of its response.

To implement a winner-takes-all system that results in only one neuron active at each location, inter-directional inhibition is implemented. This inhibition is defined between neurons selective to different directions at the same spatial location,

$$\gamma_{x,y,\theta }^{}=\sum_{\phi \neq \theta }v_{x,y,\phi }^{\text{ig}},$$ (13)

where *ϕ* indexes the preferred directions of other neurons. Another source of inhibition comes from distant neighboring neurons selective to other directions to facilitate the propagation of motion signals in the network and assist terminators to win the competition and overcome ambiguous information,

$$\zeta_{x,y,\theta }^{}=\sum_{\phi \neq \theta }\sum_{i\in \Phi }\sum_{j\in \Phi }v_{x+i,y+j,\phi }^{\text{ig}},$$ (14)

where *ϕ* is the set of locations in the effective area of long-range inhibition shown in Fig 4A. These neurons receive inhibitory connections from neurons selective to all directions *ϕ* that are three positions away, except for the neurons with the same directional preference *θ*. These inhibitory connections between integration neurons are enforced with a time delay of 60ms compared to excitatory connections. This provides sufficient time for MT neurons to propagate the motion information through the excitatory connections before suppression of their activity by inhibitory connections.

### MT segmentation neurons

The main role of segmentation neurons is detecting discontinuities in the input stimulus. The inclusion of segmentation neurons in the model is essential to control the activity of integration neurons and prevent the propagation of their activity beyond the edges of the moving stimulus. They do this by imposing an inhibitory signal upon integration neurons with the same spatial location and directional tuning. The overall dynamic behavior of these neurons is described by

$$\frac{dv_{x,y,\theta }^{\text{sg}}}{dt}=h\left(G_{\text{sg}}^{\text{cx}}v_{x,y,\theta }^{\text{cx}}-G_{\text{sg}}^{\text{es}}v_{x,y,\theta }^{\text{es}}+G_{\text{sg}}^{\text{ig}}\eta_{x,y}+G_{sg}^{sg}\chi_{x,y,\theta }^{\text{e}}-G_{\text{sg}}^{\text{sg}}\chi_{x,y,\theta }^{\text{i}}-\tau_{\text{sg}}v_{x,y,\theta }^{\text{sg}}\right),$$ (15)

where *η*_*x*, *y*_ is the excitatory input received from integration neurons and *χ*_*x*, *y*, *θ*_ represents the excitatory and inhibitory interconnection between segmentation neurons as the result of center-surround interactions. All of the remaining parameters are constant and their values are given in Table 2.

The input to segmentation cells is provided by complex neurons in V1. The segmentation neurons also become active when there is more than one moving object at the same spatial location. To implement this, they receive excitatory input from integration neurons selective to different directions at the same location. This also helps the winner-takes-all system of the MT integration neurons, as the segmentation neurons in turn provide inhibitory input to other integration neurons. This interaction is defined by

$$\eta_{x,y}=\sum_{\theta =1}^{8}v_{x,y,\theta }^{\text{ig}}\text{.}$$ (16)

To detect discontinuities in the input image, the inhibition is defined based on center-surround receptive field antagonism. As a result, the activity of neurons is suppressed when the motions at the center and surround of the neuron’s receptive field are in the same direction,

$$\chi_{x,y,\theta }^{}=\left\{ \begin{array}{l} \sum_{i\in \Pi }\sum_{j\in \Pi }v_{x+i,y+j,\theta }^{\text{sg}},\text{ if }v_{x,y,\theta }^{\text{sg}}>\rho_{\text{sg}}\text{ and }v_{x+i,y+j,\theta }^{\text{sg}}>\rho_{\text{sg}}\text{, }\\ 0\text{, otherwise,} \end{array}\right.\;$$ (17)

where Π indicates the surround of an MT neuron (Fig 4B). The receptive fields of these neurons are modeled by Gaussian filters. The spatial extent of the receptive fields of MT neurons is larger than V1 neurons. The center of the receptive field of these neurons covers 7 pixels of the stimulus and the spatial extent of the surround is 10 pixels of the stimulus. This surround suppression is effective when motion in the center and surround are in the same direction if the neuron’s activity is above the threshold, *ρ*_*sg*_ (Fig 4C and 4D). The value of the surround suppression depends on the level of activity of segmentation neurons located in the receptive field’s center.

The other source of inhibition for segmentation cells is end-stopped neurons in V1. The presence of segmentation neurons limits the propagation of motion signals. Therefore, segmentation neurons must receive inhibition from end-stopped cells to prevent interference with the propagation of accurate motion signals from the end-points of the stimulus. Spontaneous activity is defined for segmentation neurons to prevent the propagation of the activities of the neurons to other regions of the image where segmentation neurons are inactive because of a lack of motion.

### Parameter optimization

The values of the parameters used in the model as weights of the excitatory and inhibitory connections are optimized using a genetic algorithm [27]. A genetic algorithm is a heuristic method that resembles ‘natural selection’, where the fittest members of a population survive and reproduce most effectively through successive generations. This method is generally used for optimization and search problems. A rough estimation of the value of the parameters is used as an initial population for the genetic algorithm. Following this, each string is evaluated and assigned a fitness value (cost function), *F*(*θ*), which is computed based on the activity levels of the MT integration and segmentation neurons and complex neurons in V1 according to the following equations:

$$F(\theta )=F_{1}(\theta )+F_{2}(\theta )$$ (18)

$$F_{1}(\theta )=\sum_{x}\sum_{y}\left((1-\sum_{\phi \neq \theta }v_{\text{x},\text{y},\phi }^{\text{ig}})v_{x,y,\theta }^{\text{ig}}\;v_{x,y,\theta }^{cx}\right)+\sum_{x}\sum_{y}\left((1-\sum_{\phi }v_{\text{x},\text{y},\phi }^{\text{ig}})\left(1-v_{x,y,\theta }^{\text{cx}}\right)\right)$$ (19)

$$\begin{array}{ll} F_{2}(\theta )= & \left(\sum_{x}\sum_{y}v_{x,y,\theta }^{\text{sg}}\;v_{x,y,\theta }^{\text{cx}}\;\left(1-\left(\frac{1}{8}\sum_{i=-1}^{1}\sum_{j=-1}^{1}v_{\text{x}+\text{i,y}+\text{j,}\theta }^{\text{cx}}-v_{x,y,\theta }^{\text{cx}}\right)\right)\right)\\ & +\sum_{x}\sum_{y}(1-v_{\text{x,y,}\theta }^{\text{sg}})\;v_{x,y,\theta }^{\text{cx}}+\sum_{x}\sum_{y}(1-v_{x,y,\theta }^{\text{sg}})(1-v_{x,y,\theta }^{\text{ig}}). \end{array}$$ (20)

The goal of the algorithm is to maximize the value of the cost function by iteratively adjusting the parameters, $G_{\text{ig}}^{\text{cx}}$, $G_{\text{ig}}^{\text{es}}$, $G_{\text{sg}}^{\text{es}}$, $G_{\text{ig}}^{\text{ig2}}$, $G_{\text{ig}}^{\text{ig1}}$, $G_{\text{ig}}^{\text{sg}}$, $G_{sg}^{sg}$, $G_{\text{sg}}^{\text{cx}}$, $G_{\text{ig}}^{\text{ig3}}$, $G_{\text{sg}}^{\text{ig}}$, and *τ*_ig/sg_. The value of the fitness function, *F*_*1*_, depends on the activity of the neurons where there is motion, expressed by the first term in Eq (19). This term reaches its highest value when complex V1 neurons selective to direction *θ* are active in response to the stimulus moving in direction *θ*. A complex V1 neuron is considered active when its level of activity is higher than 0.01. In this case, it is expected that integration neurons at the same location, which are selective for direction *θ*, have a high level of activity and those selective to other directions *ϕ*, are inactive. An integration neuron is considered to be inactive when the level of activity is below the threshold 0.15. The second term in Eq (19) depends on the activity of neurons outside the border of the moving stimulus where there is no motion. This term has a high value when integration neurons have a very low activity in the background area, where the complex V1 neurons are not active.

The fitness function, *F*_*2*_, is based on the activity of the segmentation neurons. The first term in Eq (20) depends on the activity of segmentation neurons at the discontinuities and has a high value when segmentation neurons are active at these locations. Discontinuities of the input stimulus are expressed by the activity of complex V1 neurons where they have a high level of activity at the borders, while most of their neighboring V1 neurons are inactive. The second term in Eq (20) relates to the coherent part of the stimulus, where complex V1 neurons are active while their corresponding segmentation neurons have a low level of activity. The third term depends on the activity of the segmentation neurons in the background, where there is no motion. This term has a high value when neurons at these locations are not active.

The selection process is performed based on the value of the fitness function of the current population. The sets of parameters that result in higher values of the fitness function will be selected as the next generation. The next generation of offspring is produced by applying the following three common operators in the genetic algorithm to the population that has a higher fitness value, as follows.

Reproduction is applied to create 10% of the new population. Crossover is applied to create 50% of the new population. In the crossover operation, portions of the data are swapped between two parent members of the population with high values of the fitness function. The mutation operator is applied to the remaining 40% of the population to introduce diversity, and is applied to individual population members. We use a simplified version of Gaussian mutation to maintain diversity in the population [28].

To decrease the computational load, a combination of the genetic algorithm and the following heuristics are applied, which use some basic information about the expected dynamic behavior of the neurons to adjust the parameters. This modification is applied on the mutation operator; instead of sampling from a normally distributed random variable, a uniformly distributed random function is used in which the sign depends on the measures below. Applying these conditions to the genetic algorithm restricts the area of the search and speeds up the process by which the algorithm finds solution regions. This is performed based on the following three evaluations of the activity of MT neurons,

1. **The ability of integration neurons to overcome the aperture problem with the current parameters.** In this model, it is essential that end-stopped neurons have a stronger influence than complex neurons for the aperture problem to be solved. When the model is not capable of dealing with the aperture problem, it is a sign that the strength of the connections from the end-stopped neurons need to be increased compared with complex V1 neurons. When the model is capable of dealing with the aperture problem but the activity of the neurons selective to other directions is above the threshold, then the gain of the inter-directional inhibition is not strong enough. Therefore, the effectiveness rate of inter-directional inhibition is increased.
2. **The activity of the neurons in spatial locations where there is no motion.** When the activities of the integration neurons propagate to the background of the image, the level of excitation between neurons is decreased while the strength of the inhibitory connection from segmentation neurons is increased to prevent the excessive propagation of the activity of the integration neurons to regions with no motion.
3. **The activity of the segmentation neurons.** As discussed above, the role of segmentation neurons is to detect discontinuities in the input image. Therefore, they must have high levels of activity at the edges independent of their preferred direction and low activity when there is no motion or there is coherency in the input stimulus. To facilitate the algorithm’s ability to find the desired parameters, a condition is set based on a threshold of the activity of the segmentation neurons to evaluate the activity of the neurons where there is coherence. In particular, the strength of the surround inhibition is increased when the activities of the segmentation neurons are above the threshold set for the coherent part of the input stimulus.

The genetic algorithm terminates when the algorithm has one or more solutions. This is indicated by the defined error, *E*^*GA*^, between the expected activity of the neurons and the activity of the neurons with the estimated parameters,

$$E^{\text{GA}}=\sum_{x}\sum_{y}\left\{ \begin{array}{l} 0\text{ if }{v_{\text{x},\text{y},\phi }^{\text{ig}}|}_{\phi \neq \theta }<T\text{ and }v_{x,y,\theta }^{\text{ig}}>T\;\\ \text{0 if }{v_{\text{x},\text{y},\phi }^{\text{ig}}|}_{\phi \neq \theta }<T\text{ and }v_{x,y,\theta }^{\text{ig}}<T\text{and }v_{x,y,\theta }^{\text{cx}}<T\\ 1-v_{x,y,\theta }^{\text{ig}}(v_{x,y,\theta }^{\text{cx}})+\;v_{x,y,\theta }^{\text{ig}}(1-v_{x,y,\theta }^{\text{cx}})+\sum_{\phi \neq \theta }(v_{\text{x},\text{y},\phi }^{\text{ig}})\text{ otherwise.} \end{array}\right.$$ (21)

The first term of the equation assigns zero error if activity of integration neurons along the stimulus selective to correct direction, *θ*, have an activity level above the threshold, *T*, and those selective to other directions have an activity level below *T*. The second term assigns zero error where the activity levels of the neurons are below *T* in the background area. The third term is computed from the relative differences between the activity levels of the neurons with the desired directions with those selective to other directions, or at locations where there is no motion. When the overall value of the error over different directions and locations for a member of the population is zero (i.e., *E*^*GA*^ = *0*), the member of the population can generate the desired activity of the model neurons. Otherwise, more evolution is necessary to find the solutions.

## Results

### Parameter optimization

**Fig 5:**
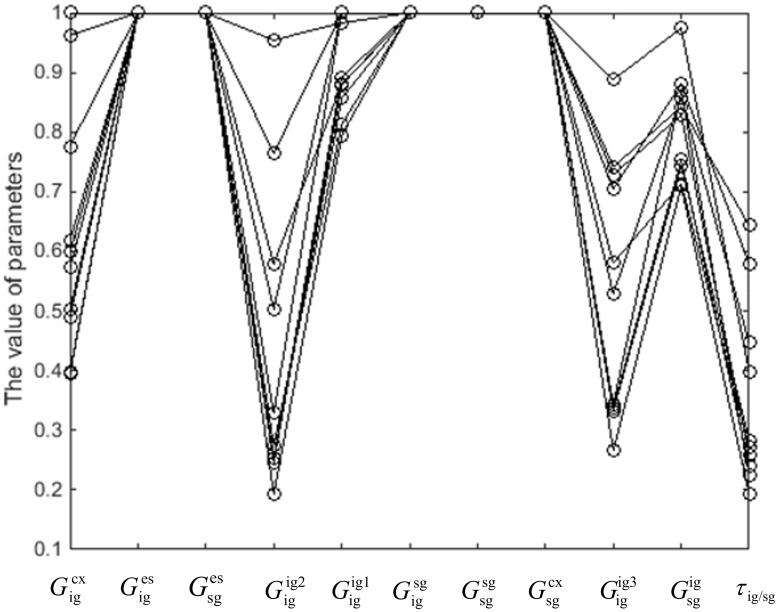
Results of estimation of the constant parameters in the model using the Genetic Algorithm (GA). Each line represents a different set of parameters. The optimization algorithm has successfully converged to the solution for different sets of parameters. The model is not very sensitive to small changes in the values of some parameters.

Fig 5 shows the results of the genetic algorithm for a population with 100 members. Different sets of parameters that resulted in zero error, *E*, are plotted. The weights of connections from end-stopped neurons to integration neurons,$G_{\text{ig}}^{\text{es}}$, are always higher than the weights of connections from complex neurons to integration neurons, $G_{\text{ig}}^{\text{cx}}$. The weights of excitatory connections between neurons can take different values but are all above a certain threshold. However, there is less variation between the weights of inhibitory connections and the weights of connections between segmentation neurons.

### The responses of neurons in V1 and MT

**Fig 6:**
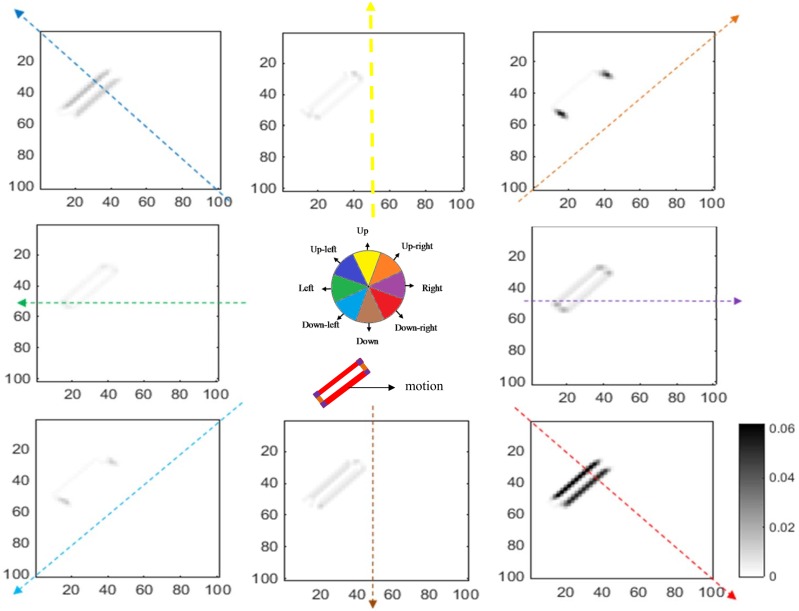
The activity of model V1 complex neurons. Each graph shows the activity of the V1 complex neurons selective to the direction shown by the colored arrow. The angle of each arrow also indicates its direction. The axes represent the location and the gray scale intensity indicates the level of activity. Neurons at the edges have higher activity compared to neurons at the terminators, which have unambiguous motion signals. The cartoon in the middle summarizes the results shown in eight graphs. The colored section of the bar shows neurons selective to the directions that have the highest levels of activity at those locations. For a bar moving towards the right, the terminators, indicated by the purple color, show the correct direction of motion; the colors of the edges represent the directions that are incorrect because of the aperture problem.

The responses of complex neurons in V1 to a single bar oriented at 45°, moving to the right, are shown in Fig 6. The figure illustrates activity levels for neurons at different locations when the bar is located at a particular point along its trajectory. The level of activity is represented by the grey shaded areas (note the color bar). The neurons located along the bar are activated by the direction of motion perpendicular to the edges (in this case, mainly up-right and down-right), while neurons at the endpoints have unambiguous, correct estimates of motion. It is also important to note that the activities of neurons along the edges of the bar are much stronger than the activities of neurons at the terminators.

**Fig 7:**
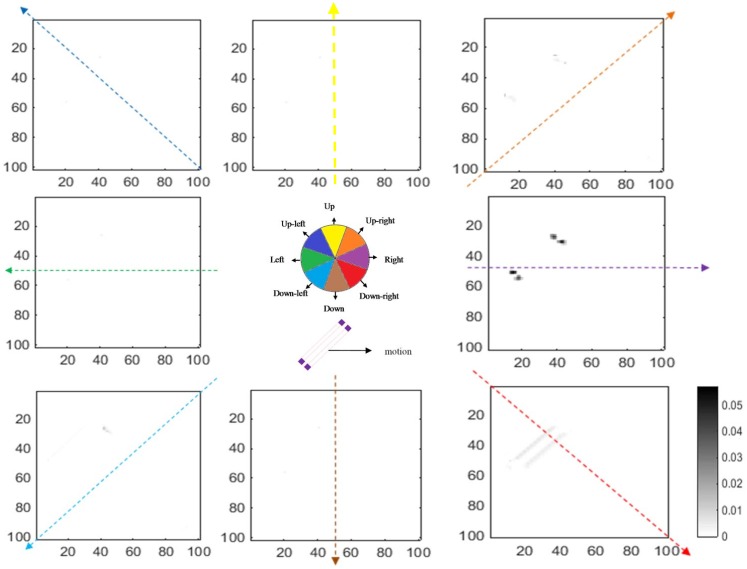
The responses of model end-stopped neurons in V1 (plotted using the same format as in Fig 6). The neurons at the end-points of the bar have much stronger activity compared to neurons along the edge. In this case, the input stimulus is a bar moving to the right, so neurons at the terminators selective to this direction have higher activity. As a result of lateral inhibition between neurons in V1, the activities of the neurons along the bar are suppressed.

The activity levels of end-stopped neurons in V1 for the same angled bar moving towards the right are shown in Fig 7. The activities of neurons representing ambiguous directions of motion are reduced as a result of the lateral inhibition between end-stopped neurons. Therefore, neurons at the terminators selective for rightward motion have the highest level of activity compared to other neurons.

**Fig 8:**
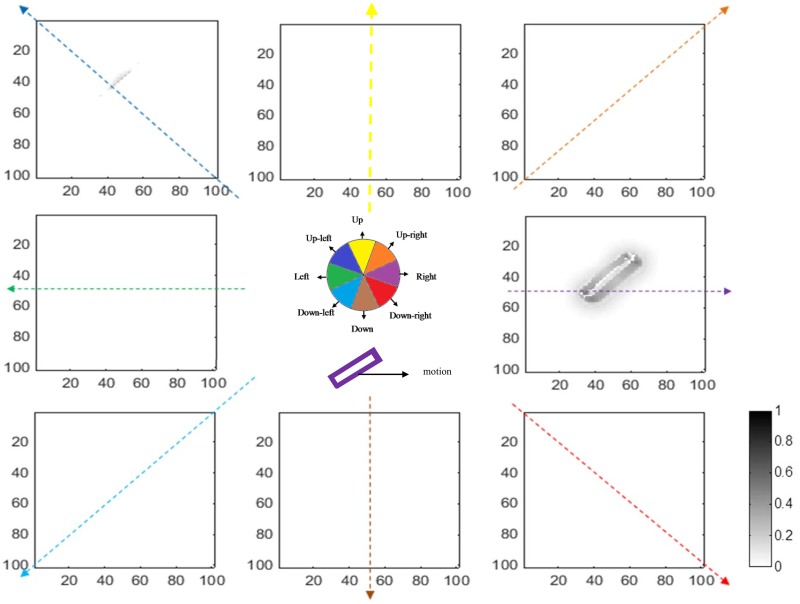
The activities of integration neurons for a stimulus moving to the right (plotted in a format similar to Fig 6). In this case, neurons representing the location of the bar, which are selective for motion to the right, have the highest levels of activity. The cartoon in the middle summarizes the results shown in the eight graphs. Neurons selective for rightward motion (purple) have the highest activity in the locations where there is motion.

**Fig 9:**
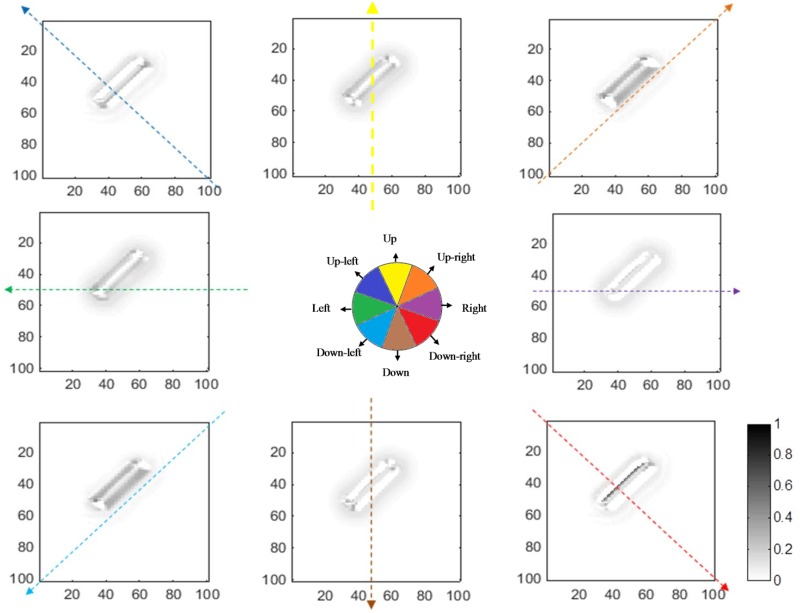
The activity levels of segmentation neurons for a stimulus moving to the right (plotted in the same format as Fig 6). As expected, these neurons have a high level of activity at the discontinuities of the input image.

The final outputs of the model, which are the responses of the MT neurons for the same stimulus, are presented in Figs 8 and 9. The results in Fig 8 show that all of the integration neurons selective to the rightward direction have a high level of activity. This means that integration neurons have successfully integrated the local motion signals and they represent correct estimations of the direction. On the other hand, segmentation neurons, shown in Fig 9, have high levels of activity at the edges of the moving bar, indicating the discontinuities in the input image.

### The role of end-stopped neurons in motion perception

**Fig 10:**
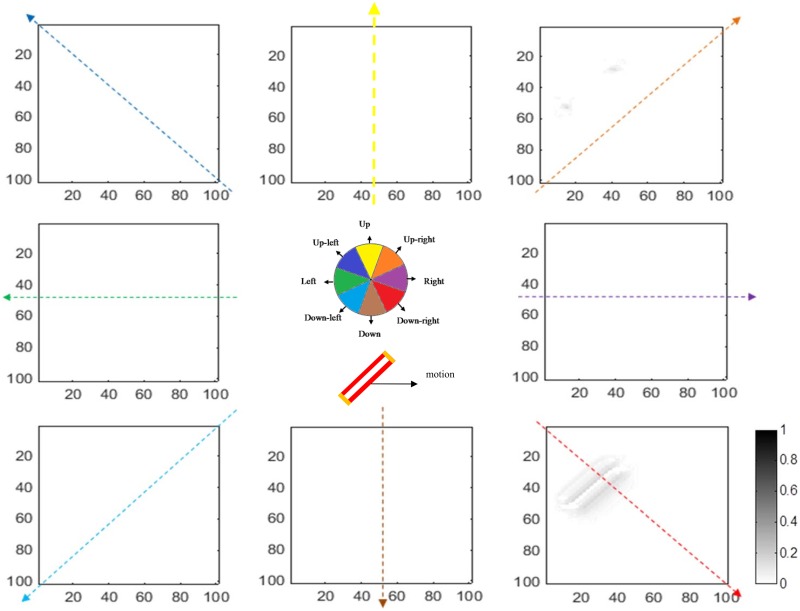
The activities of MT integration neurons when they do not receive input from end-stopped neurons, when the input stimulus is a bar moving to the right (plotted similarly to Fig 6).

To illustrate the role of end-stopped neurons in the perception of motion, we modify the model such that MT neurons only receive input from complex V1 neurons that do not have end-stopped properties. Fig 10 shows the resulting activity of MT integration neurons. As a result of the aperture problem, ambiguous motion signals are propagated through the entire network and the dominant output after a certain period of time is the direction perpendicular to the edges of the bars.

**Fig 11:**
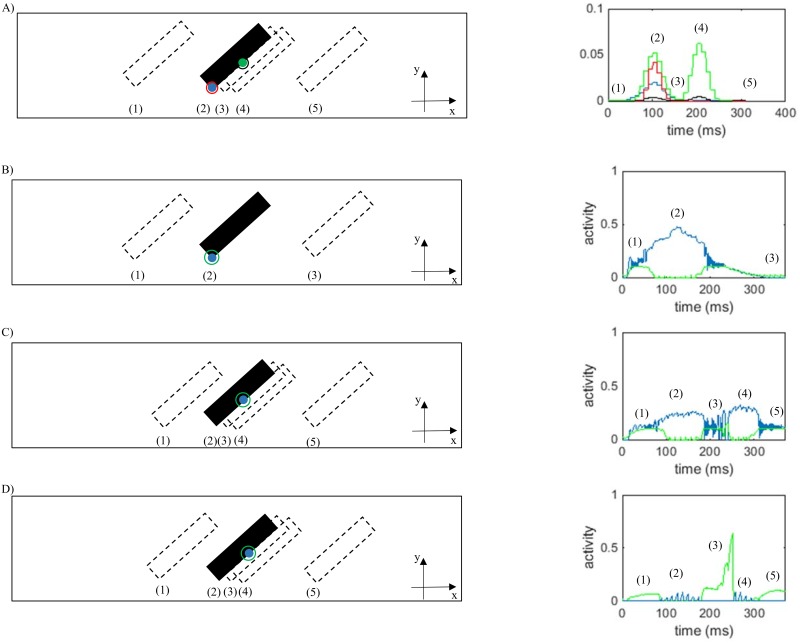
Activity of V1 neurons and MT integration and segmentation neurons in response to a bar moving to the right. The spatiotemporal location of the bar is indicated by (1), (2), (3)… A) The activity of the complex V1 neuron selective to rightward motion at the terminator (blue), the activity of a complex V1 neuron selective for downward-right motion along the bar (green), the activity of an end-stopped neuron selective to rightward motion at the terminator (red), and the activity of an end-stopped neuron selective to downward-right motion along the bar (black). B) The activity of an integration neuron (blue) and a segmentation neuron (green), both selective to rightward motion and located at the terminator. C) The activity of an integration neuron (blue) and a segmentation neuron (green) selective to the rightward-down direction located at the middle of the bar. D) The activity of an integration neuron (blue) and a segmentation neurons (green) selective to rightward-down motion located at the middle of the bar (green).

To further examine the necessity of end-stopped neurons in the model, the activity of a complex V1 neuron selective for rightward motion at the terminator is compared to the activity of the neurons along the bar selective to right-downward motion, shown in Fig 11. Among the neurons selective for motion to the right, those located at the terminators representing the correct direction of motion have the highest activities. To overcome the aperture problem, the activity of these neurons should win the competition over the neurons with ambiguous information.

Fig 11A shows the dynamic behavior of V1 neurons. The selected complex neuron (blue) does not respond when the bar has not reached the receptive field of the neuron (location 1) and its activity increases as the bar enters the receptive field of the neuron (location 2). The activity of the neuron gradually decreases as the bar passes this point (location 3). The activity of the neuron along the bar selective to downward-right motion (green) is much higher than the activity of the neuron located at the terminator (blue). Therefore, the neuron preferring rightward-down motion wins the competition over the neuron at the terminator, as it has a higher level of activity. The end-stopped neurons need to be added to the model. These neurons respond strongly to the motion of the terminators. As shown in Fig 11A, the activity of an end-stopped neuron selective to the correct direction of motion at the terminator (red) is higher than the activity of an end-stopped neuron along the edge (black). Therefore, with a higher activity for end-stopped neurons, those at the terminators will win the competition over complex neurons.

Fig 11B shows the dynamic behaviors of integration and segmentation neurons over time. The activities of the neurons increase as the stimulus enters the receptive fields of neurons within MT due to the propagation of activity via the recurrent connections. The activity of the integration neuron indicated by the blue dot increases to a maximum when the corner of the object reaches the receptive field of the neuron (location 2) and then decreases gradually as the bar passes beyond that location. The activity of the segmentation neuron, whose location is indicated by the green circle, starts to increase as the edge of the bar moves closer to the receptive field of the neuron (location 1). It then decreases because it receives inhibitory inputs from end-stopped neurons. This reduction in the activity of segmentation neurons facilitates the propagation of activity from the terminator along the object.

Fig 11C shows activity of the integration and segmentation neurons located in the middle of the bar. The activity of the integration and segmentation neuron selective to the rightward direction increases as the bar gets closer to the center of the receptive field of the neurons. The increase in the activity of the segmentation neuron is because of the perception of the discontinuity in the input stimulus (location 1). As the edge of the bar passes through this point, the activity of the integration neuron selective to rightward motion increases with a decrease in the activity of segmentation neurons (location 2). This dynamic behavior in the neurons selective to the rightward motion coincides with the changes in the activity of integration and segmentation neurons selective to rightward-down motion over time (Fig 11D). The activity of the integration neuron selective to rightward-down motion drops with an increase in the activity of the integration neuron selective to the rightward motion due to the inhibition received from neurons with unambiguous motion signals. The small fluctuation in the activity of the integration neuron selective to rightward-down motion is due to the competition between neurons selective to different directions over time (location 2 in Fig 11D). The activity of integration neurons selective to rightward motion drops slightly when the coherent section of the bar is in the receptive field of the neuron (location 3 in Fig 11C), and rises again as the second edge of the bar passes through the receptive field of the selected neuron. As the edge of the bar moves closer to the receptive field of the neuron, the competition between integration neurons selective to the correct direction of motion and those with ambiguous information commences, which gives rise to the fluctuation in the activity of the integration neuron and an increase in the activity of the segmentation neuron that detects the edges of the bar (location 4 in Fig 11C). The activity of the segmentation neurons increases when there is a discontinuity in the input stimulus (location 5 in Fig 11C and 11D).

The required time for propagation of the activity from the terminators along the whole of the object can be roughly estimated by means of the temporal dynamic shown in Fig 11. However, this time depends on the length of the bar. The overall simulation time that is assumed for MT neurons is 12ms with the time step of 0.1ms. It takes less than 12ms for the MT neurons stimulated with a shorter bar to propagate the activity from the terminators through the whole of the object and inhibit the activity of the neurons selective to other directions. The simulations are performed with a stimulus moving at one pixel per frame. The image information of the next frame is transmitted to the model after around 20ms.

### Quantitative evaluation on the robustness of modeled MT neurons

The robustness of the model in response to a single bar with different orientations and lengths and moving in different directions is evaluated. An error in a neural response at a particular pixel is considered to occur when any integration neuron that represents a wrong direction of motion has higher activity than the neuron that represents the correct direction. The model is deemed to give a correct overall response when there are more integration neurons responding to the correct direction than to a wrong direction. Therefore, the overall response error is

$$E=\{\begin{array}{ll} 0 & \text{if for all }\varphi \neq \theta ,\;\sum_{x}\sum_{y}C_{x,y,\theta }>\sum_{x}\sum_{y}C_{x,y,\varphi }\\ 1 & \text{otherwise,} \end{array}$$ (22)

where *C =* 1 is assigned to the winning neuron and *C =* 0 is assigned to the other neurons at each location. The coordinates (*x*, *y*) that are considered are those in the vicinity of the moving object, where integration cells are expected to respond, plus three neighboring neurons. The error is zero if the spatial summation of *C* is higher for the correct direction, *θ*, than the other directions, *φ*.

The activities of the MT neurons are considered for the single bar stimulus with different directions of movement, angles of orientation, and sizes of the bar. In particular, the model is tested with motion to the right, left, up and down, with orientations 0, 45, 90, and 135 degrees, for short and long bars, and with narrow and wide bars. The results demonstrate accurate performance and robustness of the model MT neurons in response to these different stimuli with the zero error in all cases.

To further evaluate the robustness of the model, stochastic noise is added to the outputs of the neurons in areas V1 and MT. The noise has a Gaussian distribution with variance depending on the level of output activity of the neuron to model neural spiking activity. For example, the output of an integration neuron at position (*x*, *y*) and direction *θ* is modified as

$${\hat{v}}_{x,y,\theta }^{ig}=v_{x,y,\theta }^{\text{ig}}+\alpha N(0,v_{x,y,\theta }^{\text{ig}}),$$ (23)

where *N*(*μ*, σ^2^) is the normal distribution with mean *μ* and variance σ^2^. The parameter *α* is used to scale the noise, with *α* = 0 giving no noise and *α* = 1 corresponding to Poisson spiking statistics. We investigated the impact of *α* varying from 0 to 1 to evaluate robustness of the response of the neurons to a stimulus that is a single short bar of 45 degrees orientation moving to the right. The error was obtained for 10 repeats with 11 different values of *α* for a single frame of the input stimulus (bar in the middle of the frame), and the mean and standard deviation of the errors were calculated. The value, *α* = 1, is an extreme level of noise—vision neurons generally fire more regularly than Poisson statistics [29,30]–but noise up to this level has been included to illustrate the network’s performance over a wide range of noise levels.

**Fig 12:**
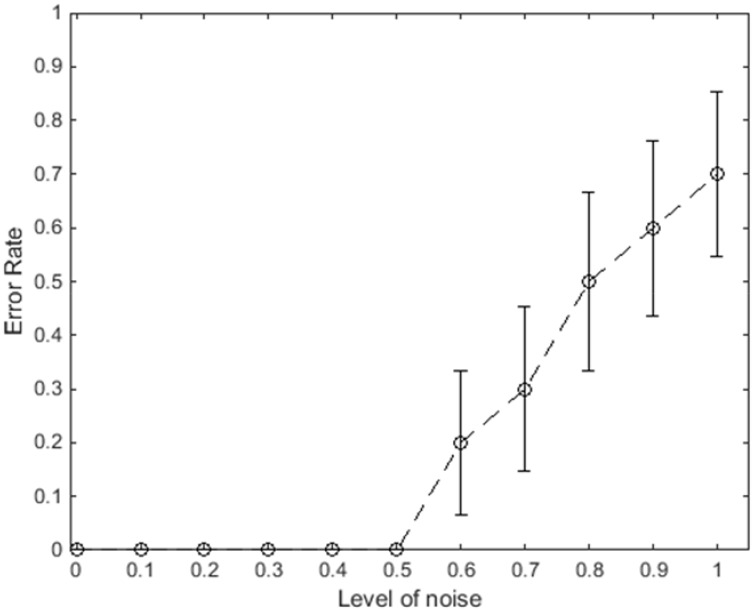
The average error of the integration neurons in the network to correctly classify the direction of motion with different levels of neural noise. The represented error is the average of the measured values of the error after 10 experiments and the error bars indicate standard error of the mean. An error of 0 represents an accurate estimation of motion by a majority of the MT neurons while an error of 1 indicates that the majority winning MT neurons have wrong estimates of the direction of motion, measured in a region within three pixels of the edges of the moving bar in one frame of the motion.

Fig 12 shows the mean error with different levels of the noise, *α*, at the outputs of the neurons; the error bars show the standard error. The results show that the model is robust in estimating the correct direction of motion up to *α* = 0.5. The error grows with further increases in the noise level. All of the errors made are confusions caused by the aperture effect, where the majority of the neurons indicate movement down-right. At *α* = 0.8, the network performance is at chance level, where around half of the repeats result in correct direction of motion and the other half result in direction influenced by the aperture effect. Further increase of *α* leads to further dominance of the aperture effect.

## Discussion

In our model, the process of motion perception is performed in two steps. The initial motion signals are generated by neurons with receptive field properties similar to those found in V1. The outputs from these neurons are processed by a model that replicates the receptive field characteristics of MT neurons. The interconnections between MT integration neurons propagate unambiguous motion signals from the termination points along the whole of the moving object and suppress ambiguous information that results from the aperture problem. MT segmentation neurons detect discontinuities in the input stimulus and prevent the propagation of motion signals from the edge of the object to the background of the image where there is no motion.

Our model suggests that overcoming the aperture problem is a two-stage process that, based on known physiological properties, likely involves neurons in area MT and special-complex neurons in V1 that have end-stopped receptive fields [20]. The outputs of the model V1 complex cells show that the end-points of a moving bar contain information that allows a calculation of the correct estimation of motion. The problem is that the end-points only cover a very small area of the input stimulus and a large proportion of the motion signals extracted by V1 neurons are ambiguous. To propagate the unambiguous information from the terminators and suppress the ambiguity, interactions between neurons in the second stage of the model (proposed in MT) are necessary. These neurons integrate the received local motion signals from V1. Therefore, the process of estimation of the direction of motion is temporally dynamic [31].

As well as input from V1 complex neurons, to overcome the aperture problem, the model uses the activity of end-stopped neurons as input to the model of MT neurons, and excitation from neighboring neurons with similar direction selectivities. This allows the activity of neurons at the terminators, which only cover a small area of the input, to suppress the activity of the neurons along the object, which covers a larger area of the input stimulus.

There is also neurophysiological evidence that some of the neurons projecting to area MT are end-stopped. Movshon and Newsome (1996) showed that these neurons respond optimally to bars that are shorter than their receptive fields. These neurons are also directionally selective and respond to the component motion of plaids rather than pattern motion [20].

Apart from the biologically plausible structure of our proposed model, the results demonstrate the robustness of the model to a large amount of noise. This is because of the low-pass filtering of the neural update equations and the recurrent connections between neurons. Gur et al. (2000) showed that the coefficient of variation (CV) in V1 cells had an average of 0.18 and has a maximum of 0.4 in alert monkey [29]. This corresponds to the noise scaling parameter, *α*, less than 0.2, which is well within the range where performance was robust in the network model. Thus, the activities of a few end-stopped neurons (at the corners of the moving bar) are sufficient to overcome the effects of expected levels of neural output noise.

Our model is inspired by a model proposed by Liden and Pack (1999) who computed the correlation between two consecutive image frames as the initial motion signals [14]. They used this method because of its simplicity. However, their model of V1 neurons had a maximum level of activity when there was no motion; i.e., the cells responded maximally to the stationary background of the image. This is not only biologically unrealistic, but also causes difficulties at the next stage of computation because of the excitatory connections between neurons. There is also no mechanism to differentiate the unambiguous activity of the neurons at the terminators from the activity of the neurons along the edges. Therefore, in the competition between neurons those regions that have a high density of active neurons (i.e. along the edges) win the competition over other neurons.

A model proposed by Beck and Neumann (2011) used a similar scheme. However, the principle for dealing with the aperture problem in their model is feedback connections from MT neurons with larger receptive fields than V1, which contradicts neurophysiological finding by Pack and Gartland (2004) [32,33]. In our model, the propagation of the unambiguous motion signals from the terminators results from recurrent interconnection between excitatory neurons.

In the 3D formation model proposed by Grossberg and Mingolla (2001), the role of end-stopped neurons is mainly in the form processing stream, which results in spatial sharpening of the neuron’s activity. In their model, it is not clear how the unambiguous motion information provided by these neurons directly assists the visual system to achieve accurate motion perception when there is no occluder in the input stimulus [10–12]. Our model demonstrates that end-stopped neurons have a key role in solving the aperture problem and their role is essential even where there is no illusion resulting from occlusion. In contrast to other previous work [10–12], our model also shows that solving the aperture problem does not necessarily require feedback connections from a higher-order area (e.g. MST).

The results of experiments performed by Majaj and Carandini (2007) show that MT neurons do not simply combine all of their inputs in their receptive fields and that the integration of motion in MT neurons is local [34]. In contrast to the models that compute global motion in the visual field, our proposed model estimates the corresponding motion at each spatial location, consistent with recent neurophysiological data. The model proposed by Nowlan and Sejnowski (1995) computes the motion of the moving objects in the visual field at two stages [35]. In the first step, the initial motion signals are obtained by velocity detecting units that act like neurons in V1. The outputs of these units are weighted by selection units at the next stage. The structure of this model results in estimation of the global motion of the moving objects in the receptive field but it does not provide the corresponding motion at each location. A flexible method proposed by Koechlin and Anton (1999) has similar temporal dynamics to our model. Unambiguous information is propagated through lateral excitatory connections between neurons, as is the case in our model, while the connections between neurons in the model proposed by Nowlan and Sejnowski (1995) are only feedforward [35,36].

Horn and Schunck (1981) and Hildreth and Ullman (1982) proposed models where the propagation of motion signals is based on a smoothing process [37,38]. The requirement of these methods is a continuous visual field. Therefore, these methods may encounter problems when dealing with the motion of different moving objects as there is no strategy to segregate different moving objects in visual space, while our proposed model has the potential to discriminate different moving objects in the visual field through the activity of segmentation neurons. Also there is evidence that the process of motion perception is temporally dynamic and the activity of the neurons gradually changes over time to represent the correct direction of motion.

In future research, we will augment the model to overcome other illusions in response to complicated stimuli, such as bars that cross each other while moving in different directions. This case gives the illusion of an upward or downward motion of the intersection point when the bars are moving horizontally. We propose that adding some form information to the model will be necessary in these cases.
